# Supplementary figures and images for: Misophonia in the UK: Prevalence and norms from the S-Five in a UK representative sample
Source: PLoS One. 2023 Mar 22;18(3):e0282777. doi: 10.1371/journal.pone.0282777 (PMC10032546; doi:10.1371/journal.pone.0282777)

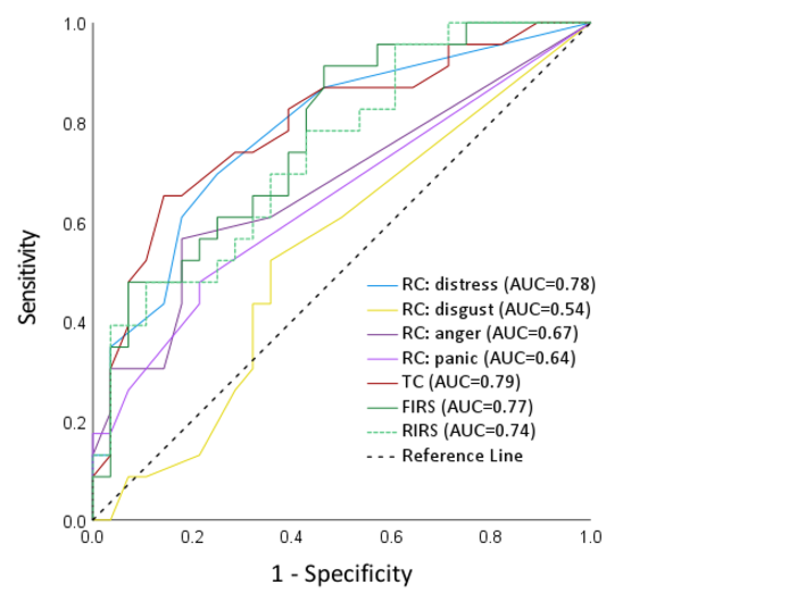

Supplement: S1 Fig — (TIF) [file pone.0282777.s001.tif]
