# Supplementary material for: Misophonia in the UK: Prevalence and norms from the S-Five in a UK representative sample
Source: PLoS One. 2023 Mar 22;18(3):e0282777. doi: 10.1371/journal.pone.0282777 (PMC10032546; doi:10.1371/journal.pone.0282777)
Supplement: S1 Table — (DOCX) [file pone.0282777.s002.docx]

# **Supporting information**

**S1 Table The SFive statements**

| **Please read each statement* carefully and base your answer on how true they feel to you based on your current thoughts, experiences, and reactions.** |
| --- |
| **Not at all true Completely true**  0 1 2 3 4 5 6 7 8 9 10 |
| **Externalising** |
| I13 People should not make certain sounds, even if they do not know about others’ sensitivities I25 I get angry at other people because of how disrespectful they are with the noises they make I06 People should do everything they can to avoid making noises that might bother others I16 I react strongly to certain sounds because I cannot stand how selfish, thoughtless, or bad-mannered people can be  I21 Certain sounds are just bad manners, and it is not strange to feel intense anger about that |
| **Internalising** |
| I18 The way I react to certain sounds makes me wonder whether deep inside I am just a bad person  I08 The way I react to certain noises makes me feel like I must be an unlikable person deep down  I05 I respect myself less because of my responses to certain sounds  I12 I feel like I must be a very angry person inside because of the way I react to certain sounds  I19 I dislike myself in the moments of my reactions to sounds |
| **Impact** |
| I20 My job opportunities are limited because of my reaction to certain noises I01 I do not meet friends as often as I would like to because of the noises they make I14 There are places I would like to go but do not, because I am too worried about how the noises will impact me  I15 I can see future where I cannot do everyday things because of my reactions to noises I09 The way I feel/react to certain sounds will eventually isolate me and prevent me from doing everyday things |
| **Outburst** |
| I17 I can get so angry at certain noises that I get physically aggressive towards people to make them stop I22 Sometimes I get so distressed by noises that I use violence to try and make it stop I23 Some sounds are so unbearable that I will shout at people to make them stop I04 If people make certain sounds that I cannot bear, I become verbally aggressive I24 I am afraid I will do something aggressive or violent because I cannot stand the noise someone is making |
| **Threat** |
| I11 I feel trapped if I cannot get away from certain noises I07 I feel anxious if I cannot avoid listening to certain sounds I02 If I cannot get away from certain noises, I am afraid I might panic or feel like I will explode I03 If I cannot avoid certain sounds, I feel helpless I10 I can experience distress as the result of some noises |
| *** Items should be randomised before being administered and without enumeration. |
