# Supplementary material for: Misophonia in the UK: Prevalence and norms from the S-Five in a UK representative sample
Source: PLoS One. 2023 Mar 22;18(3):e0282777. doi: 10.1371/journal.pone.0282777 (PMC10032546; doi:10.1371/journal.pone.0282777)
Supplement: S2 Table — (DOCX) [file pone.0282777.s003.docx]

# **Supporting information**

**S2 Table. Scoring instructions for the S-Five.**

| **Factor** | **Item number** | **Score calculation** | **Range** |
| --- | --- | --- | --- |
| Externalising | I13, I25, I06, I16, I21 | I13 + I25 + I06 + I16 + I21 | 0-50 |
| Internalising | I18, I08, I05, I12, I19 | I18 + I08 + I05 + I12 + I19 | 0-50 |
| Impact | I20, I01, I14, I15, I09 | I20 + I01 + I14 + I15 + I09 | 0-50 |
| Outburst | I17, I22, I23, I04, I24 | I17 + I22 + I23 + I04 + I24 | 0-50 |
| Threat | I11, I07, I02, I03, I10 | I11 + I07 + I02 + I03 + I10 | 0-50 |
| Total S-Five-E score | I01-I25 | $\Sigma$(I01-I25) | 0-250 |
| *Each factor has 5 items rated on a 0-10 ordinal scale, thus the scores are directly comparable in terms of statement endorsement.    The scoring guide and the programming codes (SPSS, R project, Stata) to obtain all factors and indices are freely available upon request made to the first author. The S-Five, © Copyright King’s College London, 2021. All Rights Reserved. | | | |
