# Supplementary material for: Misophonia in the UK: Prevalence and norms from the S-Five in a UK representative sample
Source: PLoS One. 2023 Mar 22;18(3):e0282777. doi: 10.1371/journal.pone.0282777 (PMC10032546; doi:10.1371/journal.pone.0282777)
Supplement: S3 Table — (DOCX) [file pone.0282777.s004.docx]

# **Supporting information**

**S3 Table. The S-Five-t triggers checklist and scoring.**

| *Trigger reaction items* |
| --- |
| **Thinking about the past few weeks, what is the main feeling this sound* has caused you?** *(please choose the most characteristic one)* |
| No feeling Irritation Distress Disgust Anger Panic Other feeling: Negative Other feeling: Positive Other: Physiological reaction |
| Sound* |
|  |
| *Trigger intensity items* |
| **Thinking about the past few weeks, please rate the intensity of your reaction to this sound* when made by another person or object** *(from 0: doesn't bother me at all to 10: unbearable/causes suffering)***.** |
| **Doesn’t bother me at all Unbearable/causes suffering**  0 1 2 3 4 5 6 7 8 9 10 |
| Sound* |
| ***List of triggers currently included in the S-Five-t:** Normal eating sounds, Certain letter sounds, Mushy foods being eaten, Sound of clipping nails, Swallowing, Keyboard tapping, Lip smacking, Normal breathing, Repetitive engine noises, Loud/unusual breathing, Mobile phone sounds, Repetitive coughing, Humming noise, Repetitive sniffing, Snoring, Certain accents, Whistling sound, Sound of tapping, Rustling, Chewing gum, Footsteps, Hiccups, Slurping, Cutlery noises, Sneezing, Certain words, Kissing, Joint cracking, Muffled sounds, Throat clearing, Baby crying, Repetitive barking, Loud chewing, Clock ticking, Crunching eating sounds, Teeth sucking, Yawning. |
| *Scoring* |
| **Trigger Count (TC)** for each participant over all triggers: the index is computed by counting the number of non-zero responses in the trigger intensity items. The index takes values between 0 and the number of triggers considered, here 37, and provides information on the volume of triggers.  *Example: if an individual selected ‘no feeling’ or, ‘other: positive’ reaction to a trigger to 32 out of 37 triggers, their TC would be 5, that is, the number of triggers they experience.*  **Reaction Count (RC)** for each trigger over all participants: the index is computed for each reaction type separately, by counting over all participants the times a certain reaction was selected (using the trigger response items). The RC for each reaction (i.e., RC-Anger, RC- Irritability etc) takes values between 0 and the number of triggers considered, here 37. The index provides information on the frequency of endorsement of each specific reaction, across participants and triggers.  *Example: if an individual selected anger as their main emotional reaction to three different triggers, and panic as their main reaction to two triggers, then their RC-anger would be 3 and their RC-panic would be 2.*  **Frequency/Intensity of Reactions Score (FIRS):** the index is computed by counting the trigger intensity items. FIRS takes values between 0 and ten times the number of triggers considered, here between 0 and 370. The index provides a combined information of the number of triggers and their intensity.  *Example: if an individual reported 5 triggers and the highest possible intensity to each (that is 10), their FIRS value would be 50. The same score would correspond to a participant who reported 10 triggers but of moderate intensity 5 to each.*  **Relative Intensity of Reactions Score (RIRS):** the index is computed by dividing the FIRS index by the trigger count TC, to derive an estimation of the intensity of the responses to triggers, relative to the number of triggers reported. RIRS takes values between 0 and 100 and provides information on the intensity of the response to triggers regardless of their number.  *Example: if an individual with FIRS equal to 50 as before reported 5 triggers with intensity 10 each, their RIRS would equal 10. But if an individual receives the same FIRS (50) by reporting 10 triggers of moderate intensity (5), their RIRS would be 5.* |
| *The scoring guide and the programming codes (SPSS, R project, Stata) to obtain all factors and indices are freely available upon request made to the first author.* *The S-Five, © Copyright King’s College London, 2021. All Rights Reserved.* |
