# Supplementary material for: Misophonia in the UK: Prevalence and norms from the S-Five in a UK representative sample
Source: PLoS One. 2023 Mar 22;18(3):e0282777. doi: 10.1371/journal.pone.0282777 (PMC10032546; doi:10.1371/journal.pone.0282777)
Supplement: S4 Table — (DOCX) [file pone.0282777.s005.docx]

# **Supporting information**

**S4 Table. Full list of administered questionnaires.**

| - **Bryant and Smith Aggression Questionnaire (BS-AQ; Bryant & Smith, 2001)** is a shorter refined version of the original AQ, with 12 items rather than 29 items, rated on a five-point Likert scale. The scale captures four aspects of aggression: physical aggression, verbal aggression, anger and hostility. Higher scores are indicative of higher levels of aggressive behaviour. |
| --- |
| - **Generalized Anxiety Disorder-7 Questionnaire (GAD-7; Spitzer et al., 2006)** screens for measures severity of generalised anxiety disorder. The questionnaire asks the rater to consider the past two weeks and rate each item on a four-point scale from ‘Not at all’ to ‘Nearly every day’. The scores for each item are totalled, with higher scores suggesting higher levels of generalised anxiety. |
| - The **Patient Health Questionnaire-9 (PHQ-9)**, from Kroenke et al. (2001), is the brief assessment of depression severity. The nine DSM-5 criteria are scored on a four-point scale, with higher scores indicating higher severity of depression. |
| - The **Work and Social Adjustment Scale (WSAS; Mundt et al., 2002)** is a simple measure of impairment in functioning, consisting of five items rated on a nine-point scale from “Not at all” (0) to “Severely impaired” (8). Higher scores on the WSAS indicate a greater level of impairment in work and social aspects of life. |
| - The **Beliefs about Emotions Scale (BES; Rimes & Chalder, 2010)** is a 12-item questionnaire on beliefs regarding the inability to accept negative emotions, and the adverse consequences of experiencing and expressing those emotions. Items are rated on a seven-point Likert scale, specifying level of agreement or disagreement. |
| - The **Mindful Attention Awareness Scale (MAAS; Brown & Ryan, 2003)** assesses a receptive state of mind, which is a core feature of mindfulness. MAAS contains 15 items measured on a six-point ordinal scale ranging from ‘almost always’ to ‘almost never’. |
| - The **Autonomous Sensory Meridian Response (ASMR-15; Roberts et al., 2019)** is a questionnaire assessing altered state of consciousness phenomena, namely autonomous sensory meridian response (ASMR), which is characterised by pleasurable tingling sensation in response to certain audio-visual stimuli, causing relaxation and euphoria. The 15-item scale is rated on a scale from 1, ‘completely untrue for me’ to 5, ‘completely true for me’, and consists of four subscales: altered consciousness, sensation, relaxation and affect. |
| - The reduced-item **Disgust Propensity and Sensitivity Scale-Revised (DPSS-R; Fergus & Valentiner, 2009)** consists of 12 items on a five-point response scale that assess how easily one is disgusted, known as disgust propensity, and how bothered a person is by their disgust, which is described as disgust sensitivity, both of which contribute to disgust reactions. |
| - The **Anxiety Sensitivity Index (ASI-3; Taylor et al., 2007)** is a shorter 18-item version of the original anxiety sensitivity index (Peterson & Reiss, 1992). It assesses anxiety sensitivity conceptualised as one’s considerations regarding misinterpretations of anxiety-related sensations. The scale measures anxiety sensitivity on physical, cognitive, and social dimensions. |
| - The **Adult Eating Behaviour Questionnaire (AEBQ; Hunot et al., 2016)** is a 35-item measure that assesses appetitive traits in adulthood. AEBQ consists of eight subscales; however, for the purpose of this study only ‘food fussiness’ subscale was implemented, which consists of five items measured on a five-point Likert scale ranging from ‘strongly disagree’ to ‘strongly agree’. |
| - The **Temperament Evaluation of Memphis, Pisa, Paris and San Diego**–**autoquestionnaire (TEMPS-A; Akiskal et al., 2005)** measures temperamental variations based on diagnostic classifications for affective temperaments, namely cyclothymic, dysthymic, irritable, hyperthymic, and anxious, and has five subscales named as such. The scale is a yes-or-no type questionnaire and consists of 39 items. |
| - The **Big Five Inventory (BFI; John & Srivastava, 1999)** is a 44-item questionnaire with a five-point Likert agreement scale, which measures one’s personality on the Big Five Factors of personality: extraversion vs. introversion, agreeableness vs. antagonism, conscientiousness vs. lack of direction, neuroticism vs. emotional stability, openness vs. closedness to experience. Those factors are further separated into personality dimensions. |
| - The **Leahy Emotional Schema Scale II (LESS II; Leahy, 2012)** is a 28-item measure with a six-point ordinal response scale that determines beliefs and attributions about emotions. The scale is divided into fourteen dimensions: invalidation, incomprehensibility, guilt, simplistic view of emotion, devalued, loss of control, numbness, overly rational, duration, low consensus, non-acceptance of feelings, rumination, low expression, and blame. |
| - The **Misophonia Questionnaire (MQ; Wu et al., 2014)** is a 34-item scale consisting of three sections that assesses misophonia regarding the presence of specific triggers, emotional and behavioural responses and its severity. The first two sections, misophonia symptom scale and misophonia emotions and behaviours scale, are rated on a five-point ordinal scale, whilst the third section, Misophonia Severity Scale, measures one’s severity of sound sensitivity on a 1 to 15-point scale, with 1 suggesting minimal sound sensitivity and 15 indicating very severe sensitivity. |
| - The **Amsterdam Misophonia Scale (A-MISO-S; Schröder et al., 2013) i**s a six-item scale that measures different facets of misophonia, namely time consumed by the condition, its impact on one’s functioning, level of distress, level of resistance, perceived control over thoughts, and avoidance behaviours. |
